# Frequency of *Porphyromonas gingivalis fimA* in smokers and nonsmokers after periodontal therapy

**DOI:** 10.1590/1678-7757-2018-0205

**Published:** 2019-04-11

**Authors:** Mariana Gouvêa Latini ABREU, Dione KAWAMOTO, Marcia Pinto Alves MAYER, Vinicius D’Avila Bitencourt PASCOAL, Karina Sampaio CAIAFFA, Elizangela P. ZUZA, Cristiane DUQUE, Gabriela Alessandra da Cruz Galhardo CAMARGO

**Affiliations:** 1 Universidade Federal Fluminense, Departamento de Odontologia, Área de Periodontia, Nova Friburgo, Rio de Janeiro, Brasil.; 2 Universidade de São Paulo, Instituto de Ciências Biomédicas, Departamento de Microbiologia, São Paulo, São Paulo, Brasil.; 3 Universidade Federal Fluminense, Departamento de Ciências Básicas, Nova Friburgo, Rio de Janeiro, Brasil.; 4 Universidade Estadual Paulista (UNESP), Faculdade de Odontologia de Araçatuba, Departamento de Endodontia, Araçatuba, São Paulo, Brasil.; 5 Universidade Estadual Paulista (UNESP), Faculdade de Odontologia de Araçatuba, Departamento de Odontopediatria e Saúde Pública, Araçatuba, São Paulo, Brasil.

**Keywords:** Periodontal disease, Smoking, Porphyromonas gingivalis

## Abstract

*Porphyromonas gingivalis* is one of the most important Gram-negative anaerobe bacteria involved in the pathogenesis of periodontitis. *P. gingivalis* has an arsenal of specialized virulence factors that contribute to its pathogenicity. Among them, fimbriae play a role in the initial attachment and organization of biofilms. Different genotypes of *fimA* have been related to length of fimbriae and pathogenicity of the bacterium.

**Objectives:**

The aim of this study was to identify 5 types of *fimA* genotype strains in smokers and nonsmokers with periodontitis, before and after periodontal therapy.

**Material and Methods:**

Thirty-one patients with periodontitis harboring *P. gingivalis* were selected: 16 nonsmokers (NS) and 15 smokers (SM). Clinical and microbiological parameters were evaluated at baseline and 3 months after periodontal treatment, namely: plaque index, bleeding on probe, probing depth, gingival recession and clinical attachment level. The frequency of *P. gingivalis* and fimA genotype strains were determined by polymerase chain reaction.

**Results:**

Type I *fimA* was detected in the majority of SM and NS at baseline, and the frequency did not diminish after 3 months of treatment. The frequency of type II genotype was higher in SM than NS at baseline. After 3 months, statistical reduction was observed only for types II and V *fimA* genotypes in SM. The highest association was found between types I and II at baseline for NS (37.5%) and SM (53.3%).

**Conclusion:**

The most prevalent *P. gingivalis fimA* genotypes detected in periodontal and smoker patients were genotypes I and II. However, the presence of *fimA* genotype II was higher in SM. Periodontal treatment was effective in controlling periodontal disease and reducing type II and V *P. gingivalis fimA*.

## Introduction

Recent advances in DNA sequencing and bioinformatics technologies have provided an overview of microbiomes associated with health and disease, thereby expanding the knowledge on putative pathogenic species. Differences between periodontitis and health now are detected at the level of phylum and genus, in addition to confirming previous association of specific species with periodontal disease, including *P. gingivalis* and *T. denticola*, which are two of the main species associated with the disease, strongly related to severe forms of periodontitis^1^. *P. gingivalis* has an arsenal of specialized virulence factors that contribute to its pathogenicity. Among them, fimbriae play a role in the initial attachment and organization of biofilms, and act as adhesins that mediate invasion and colonization of host epithelial cells^2^. *P. gingivalis* generally expresses two distinct fimbriae, called FimA and Mfa1, which are composed of polymerized FimA and Mfa1 proteins encoded by the *fimA* and *mfa1* genes, respectively^3^. The *P. gingivalis* gene *fim* cluster consists of seven genes, *fimX*, *pgmA* and *fimA-E*, encoding FimX, PgmA and FimA-E proteins, respectively^4^. Six genotypes of *fimA* (I-V) and Ib were identified in *P. gingivalis* strains, and the genotype has been especially related to fimbriae length and pathogenicity of the bacterium^4^.

Previous studies have evaluated the association between the frequency of *fimA* genotypes and periodontal health status in adults^5^ ^,^ ^6^. *P. gingivalis* was detected in 36.8% of the healthy subjects and in 87.1% of the patients with periodontitis. Among the *P. gingivalis*-positive healthy adults, the most prevalent *fimA* was genotype I (76.1%), followed by genotype V (29.7%). In contrast, most patients with periodontitis carried *fimA* genotype II (66.1%), followed by genotype IV (28.9%)^6^. These findings indicate that there are both disease-associated and non-disease-associated strains of *P. gingivalis*, and their infectious traits, which influence periodontal health status, could be differentiated based on the clonal variation of *fimA* genes^6^.

Tobacco consumption is a risk factor for periodontal disease. Smoking is associated with higher clinical attachment loss and gingival recession, reduced bone height and density, and, consequently, with increased tooth loss^5^. The mechanism by which tobacco affects the periodontal tissue is related to toxic substances such as nicotine and cotinine, which has been associated with various cellular changes that may contribute to the onset and subsequent progression of periodontal disease^7^. Tobacco use promotes several adverse effects, such as reduced gingival blood flow^8^, oxidative stress and alterations in immunoinflammatory responses, reducing the functional activity of neutrophils such as chemotaxis, glycolytic activity and phagocytosis^9^; it also impairs wound healing^10^ and interferes on bacterial acquisition and host response to colonization in biofilms^11^. The frequency of periodontopathogens was previously investigated in smokers and nonsmokers. *P. gingivalis* has been detected more frequently in the periodontal pockets of smokers (66.7% – pocket depth of 3-5 mm) in comparison with nonsmokers (52.2%), and it has been found in high levels in sites with periodontitis^12^.

Scaling and root planing is the most common and well-recognized nonsurgical periodontal therapy for promoting improvement in clinical and microbiological parameters^13^. Few studies have assessed the longitudinal clinical and microbiological evaluation of smokers undergoing periodontal maintenance therapy, and controversial results have been found when comparing smokers with nonsmokers^13^ ^,^ ^14^. A reduction of 93% in *P. gingivalis* frequency for nonsmokers, compared to 88% for smokers, was observed in subgingival sites after periodontal treatment^15^. Considering the presence of different genotypes of *P. gingivalis fimA,* Teixeira, et al.^16^ (2009) suggested an association between the genotype fimA IV and disease severity in smoker-chronic periodontitis subjects. However, no study was found evaluating the influence of periodontal treatment on the frequency of different *fimA* genotypes in smokers and nonsmokers with periodontitis. The aim of this study was to compare the frequency of different genotypes of *P. gingivalis fimA* in smokers and nonsmokers with periodontitis, before and after 3 months of nonsurgical periodontal therapy. The null hypotheses are: 1) there is no difference in the periodontal status and frequency of *P. gingivalis fimA* genotypes between smokers and nonsmokers; and 2) smoking did not interfere in the response to periodontal treatment and levels of *P. gingivalis fimA* genotypes.

## Material and methods

### Study population

Thirty-one patients (15 smokers – SM and 16 nonsmokers – NS) with positive polymerase chain reaction (PCR) for *P. gingivalis*, from a total of 48 patients (24 in each group), aged 27 to 70 years, were selected to participate in this study. All participants were recruited from the Department of Periodontology, School of Dentistry, Fluminense Federal University, Nova Friburgo, RJ, Brazil, for a period of 3 months, between 2014 and 2015. The study protocol was approved by the Research Ethics Committee of the Fluminense Federal University – Nova Friburgo, RJ (CAAE: 55894816.2.0000.5626), and registered in the clinical trials (NCT02879903). Prior to participation, the purpose and procedures were fully explained to all participants, who gave their written informed consent in accordance with the Helsinki Declaration. An initial sample size of 16 participants *per* group was chosen, considering the standard deviations from a previous study^17^, the effect size [gingival index (GI), a minimum detectable change of 10%], a power of 80%, a significance level of 5%, and a loss to follow-up rate of up to 20% of the participants.

The following inclusion criteria were observed: presence of severe generalized chronic periodontitis in at least two teeth in different arches, including bleeding when probing these sites, probing depth (PPD) ≥5 mm, clinical attachment level (CAL) ≥5 mm and radiographic bone loss involving >30% of site^18^. The participants were considered heavy SM if they smoked ten or more cigarettes a day for at least 2 years. The smoking habit was confirmed at each visit, and the individuals who stopped smoking were excluded. Former SM were not included in the control group. The exclusion criteria were: patients with systemic diseases, diabetes or osteoporosis, pregnant and lactating females, use of immune suppressive medication, phenytoin, cyclosporine, calcium channel blockers or any antibiotics or nonsteroidal anti-inflammatory drugs in the past 3 months, and any medical conditions requiring immunotherapy or people diagnosed with HIV+ or AIDS, which could interfere with the periodontium status^19^.

### Clinical examination and periodontal therapy

Prior to clinical examination, a questionnaire was applied to collect information on the smoking habits of the participants (years of cigarette consumption and number consumed daily), initially at baseline and then after a 3-month follow-up examination. An experienced periodontist (GACGC) determined the clinical periodontal parameters, including plaque index (PI), bleeding on probing (BOP), pocket probing depth (PPD), gingival recession (GR), and clinical attachment level (CAL), using a periodontal probe PCP15 (PCP-UNC15, Hu-Friedy, Chicago, IL, USA), at six sites *per* tooth on all the teeth, excluding third molars. The intraexaminer agreement of the categorical variables (PI, GI) using the kappa calculation, at the tooth level, was 0.75. Reproducibility of continuous variables (PPD, GR and CAL) was 0.70, as evaluated by the intraclass correlation coefficient (ICC).

Quadrant scaling and root planing (SRP) were performed weekly at baseline on each participant under local anesthesia, using periodontal curettes (American Eagle, Gracey Access Curettes, Missoula, MT, USA) and ultrasonic scalers (Cavitron, Dentsply, York, PA, USA). The maintenance therapy included professional plaque control and SRP in recurrent periodontal pockets monthly during the 6 months of the study. No teeth had to be extracted during therapy. A different clinician (MGLA) conducted the periodontal treatment. The examiner (GACGC) had no access to previous recordings. All participants received oral hygiene instructions for home care procedures (tooth-brushing technique, interdental cleaning and use of tongue scrapers).

### Microbiological samples

After performing the clinical measurements, the supragingival biofilm was removed with sterile gauze. Subgingival samples of each participant were taken from the sites with the deepest PPD (≥5 mm), using a sterile periodontal curette. Pooled biofilms from each site were separated in microtubes containing Tris-EDTA buffer (10 mM Tris– HCl, 0.1 mM EDTA, pH 7.5), and stored at -20°C to be analyzed microbiologically using the PCR assay.

### Microbiological evaluation – PCR primers and amplification

DNA was extracted using a protocol originally described by Sardi, et al.^19^ (2011) and quantified in a spectrophotometer at 260 nm (Genesys 10UV, Rochester, NY, USA), to obtain a standard concentration of 0.1 µg/mL, and then stored at −20°C to test subsequent PCR reactions. The detection of *P. gingivalis* and *fimA* genotypes was performed as previously described^6^ ^,^ ^19^. Primers for the 16S rRNA gene were used as a positive control and *P. gingivalis* species-specific primers were used for the *fimA* genotypes^6^. All primers were custom-made by IDT (Integrated DNA Technologies, Sintese Biotecnologia, Belo Horizonte, MG, Brazil). PCR amplification was performed with a thermocycler AmpliTherm TX96 Gradient (Axygen, Corning, NY, USA) under thermal conditions specific for each pair of primers. The PCR products were separated by electrophoresis in 2% agarose gels and Tris-borate-EDTA running buffer (8.0 pH). The molecular mass ladder (100 bp DNA ladder, Gibco, Grand Island, NY, USA) was included for running the agarose gel. The DNA was stained with 0.1 µl of SYBR Safe/mL (Invitrogen, CA, USA) and visualized under UV light (Pharmacia LKB-MacroVue, San Gabriel, CA, USA). The images were photographed (ImageMaster – LISCAP, VDS, Pharmacia Biotech, Piscataway, NJ, USA) and analyzed. The amplification reaction was performed with the following cycling parameters: initial denaturation at 95°C for 10 min, PCR cycling consisting of 40 cycles, 94°C for 30 s, 58°C for 30 s, and 72°C for 30 s, followed by an extension at 72°C for 2 min. Positive and negative controls were included in each PCR set, and in each sample process.

### Statistical analysis

The statistical analysis was performed using SPSS Statistics 17.1 (IBM Inc., Chicago, IL, USA). All variables were tested for the normality of data (Shapiro-Wilk). Variables from participant’s characteristics and clinical analyses were compared between SM and NS using the Mann-Whitney test. Comparisons between the periods of time (baseline × 3 months) considering each group (SM and NS) separately were conducted using Wilcoxon tests. Differences were considered significant when p<0.05.

## Results

### Clinical results

There was no statistical difference between SM and NS regarding age, gender and race (Table 1). Median cigarette consumption was 20 cigarettes *per* day, and the duration (median) of the smoking habit was 23 years. Comparing SM and NS, at baseline, both groups had a similar periodontal status (Table 2). There was a statistically significant reduction in the clinical parameters for the NS group, with a decrease in PI (*p*=0.004) and BOP (*p*=0.011) indexes, and in PPD (*p*<0.001) and CAL (*p*=0.007) measurements, and also for the SM group, with a decrease in PPD (*p*<0.001) and CAL (*p*=0.007), comparing baseline with 3 months after periodontal treatment. SM exhibited a higher level of PI (50% -*p*<0.001) and CAL (5.68 mm – *p*=0.041) after 3 months than NS (PI: 9.69% and CAL: 3.51 mm).

**Table 1:** Characteristics of the participants

Variables	Groups	
	Nonsmokers (NS) n=16	Smokers (SM) n=15
Age (years)†	49 (44.25-61.75)	47 (33-52)
Gender n (%)		
Female	10 (62.5)	6 (40)
Male	6 (37.5)	9 (60)
Ethnicity n (%)		
White	11 (73.3)	8 (50)
Black	4 (26.6)	8 (50)
Cigarettes *per* day†	0	20 (10-20)
Duration of smoking habit†	0	23 (10-32)

*Statistically significant differences between NS and SM groups for baseline (Mann Whitney test; p<0.05)

†Values expressed in medians (percentile 25-percentile 75)

**Table 2:** Clinical evaluation of smokers versus nonsmokers at baseline and after 3 months of periodontal therapy. Values are expressed in medians (percentile 25-percentile 75)

Variables	Groups	
	Nonsmokers (NS) n=16	Smokers (SM) n=15
PI (%)		
Baseline	46.04 (14.12-71.98)	49.24 (38-86)
3 months	9.69(5.04-25.37)*	50 (19.84-72.22)‡
BOP (%)		
Baseline	29.91 (17.71-63.15)	23.07 (12.34-64.74)
3 months	13.15 (3.54-30)*	24.6 (13.49-30.2)
PD (mm)		
Baseline	5.18 (5-5.29)	5.1 (5-5.31)
3 months	2.93 (2.65-3.81)*	3.8 (3-4.12)*‡
GR (mm)		
Baseline	0.87 (0-2.62)	2 (1-2.66)
3 months	0.5 (0-2.66)	2 (1-2.57)
CAL (mm)		
Baseline	5.9 (5-8.16)	7 (6.11-7.91)
3 months	3.51 (2.75-6.29)*	5.68 (4-6.74)*‡

*Statistically significant difference between baseline and 3 months (Wilcoxon test; p<0.05)

†Statistically significant differences between NS and SM groups at baseline (Mann Whitney test; p<0.05)

‡Statistically significant differences between NS and SM groups at 3 months (Mann Whitney test; p<0.05

### *fimA* genotype results

*P. gingivalis* was detected in 16 NS and 15 SM, and distributed according to 5 genotypes of *fimA P. gingivalis* at baseline and 3 months after periodontal therapy, as described in Table 3. Genotype I was detected in the majority of SM (60%) and NS (87.5%) at baseline, and the frequency did not diminish after 3 months of treatment (SM – 60% and NS – 93.8%). The frequency of genotypes II, III and V *fimA* was higher in SM than NS at baseline; however, a statistical difference was found only in the frequency of *fimA* genotype II for SM (93.3%) compared with NS – (47.8%) (*p*=0.017). After 3 months, statistical reduction was observed only for *fimA* genotypes II and V in SM (*p*=0.001).

**Table 3:** Distribution of 5 *fimA* types of *P. gingivalis* at baseline and 3 months after periodontal therapy

*fimA* types	Frequency in % (number of patients)	
	Nonsmokers (NS)	Smokers (SM)
	**n=16**	**n=15**
I		
Baseline	87.5(14)	60(9)
3 months	93.8(15)	60(9)
II		
Baseline	47.8(7)†	93.3(14) *
3 months	12.5(2)	13.3(2)
III		
Baseline	18.8(3)	40(6)
3 months	18.8(3)	33.3(5)
IV		
Baseline	0	0
3 months	0	0
V		
Baseline	25(4)	40(6) *
3 months	18.8(3)	0

*Statistically significant difference between baseline and 3 months (Wilcoxon test; p<0.05)

†Statistically significant differences between NS and SM groups at baseline (Mann Whitney test; p<0.05)

‡Statistically significant differences between NS and SM groups at 3 months (Mann Whitney test; p<0.05)

**Figure 1:**
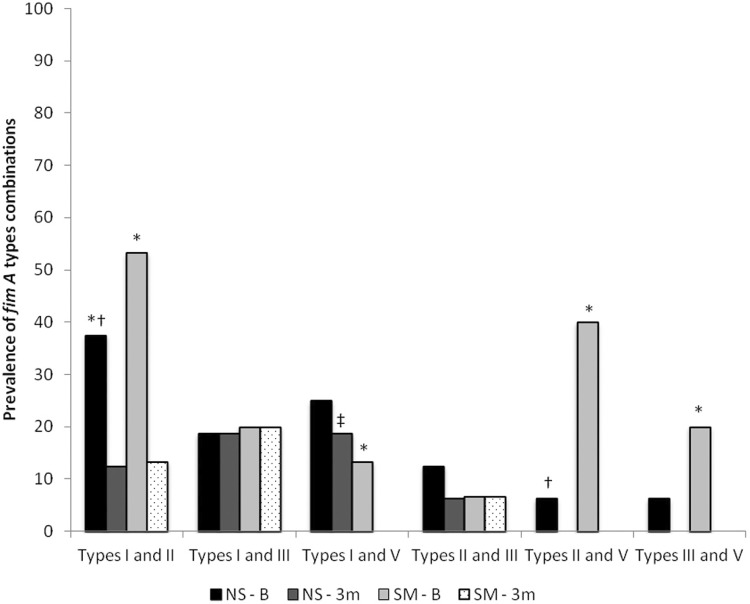
Frequency (%) of combinations of *P. gingivalis fimA* genes in smokers (SM) and nonsmokers (NS) at baseline and after 3 months of periodontal treatment. Values express percentage of *P. gingivalis* harboring *fimA* types

The frequency of two different genotypes of *fimA* in NS and SM participants, at baseline and after 3 months of treatment, are shown in Figure 1. The highest association was found between genotypes I and II at baseline for NS (37.5%) and SM (53.3%). Statistical differences were found between NS and SM, considering genotypes I and II and genotypes II and V, at baseline. The combination of genotypes I and V in SM was not detected after treatment, unlike NS. Comparing baseline with 3 months of treatment, there was a statistical reduction in the combination of *fimA* genotypes I and III for both groups. The combinations of genotypes I and V, genotypes II and V and genotypes III and V were not detected after treatment of SM participants.

*Statistically significant difference between baseline and 3 months (Wilcoxon test; p<0.05)

†Statistically significant differences between NS and SM groups for baseline (Mann Whitney test; p<0.05)

‡Statistically significant differences between NS and SM groups for 3 months (Mann Whitney test; p<0.05)

## Discussion

In this study, we confirmed that *P. gingivalis* is a common microorganism present in periodontal disease in SM and NS, corroborating other studies^16^ ^,^ ^20^. The virulence of *P. gingivalis* has been heavily associated with the presence of different types of fimbriae, which have been characterized as key factors in adhesion, invasion and colonization of this pathogen in the periodontal tissues^2^. However, the influence of periodontal treatment in the prevalence of *fimA* genotypes in SM compared with NS was not clear.

No difference was found among the groups of participants diagnosed with periodontal disease in relation to gender. Nevertheless, differences were found in literature with predominance of periodontal disease in males^21^ or females^22^. Considering ethnicity, no significant differences were found between the groups, contradicting a study that found a prevalence of periodontal disease for the black ethnic group^23^. In this study, the participants smoked an average of 20 cigarettes *per* day, characterizing them as heavy SM. A study pointed that there was greater severity of periodontal disease in patients who consumed more than 20 cigarettes *per* day, with a 10% prevalence of insertion loss in heavy SM^24^. Light SM, who consumed fewer than 4 cigarettes/day had an alveolar bone loss of 3.3 mm, whereas heavy SM had a loss of 7.3 mm, showing a direct relation between the number of cigarettes consumed and the rate of progression of periodontal disease^6^.

Some studies have shown that SM have higher prevalence and severity of periodontal disease, and worse results after periodontal therapy than NS^7^ ^,^ ^25^. In this study, SM and NS were matched by periodontal parameters (PI, BOP, PPD, GR and CAL) at the baseline, since no statistical difference was observed between the groups. Besides, instruction on oral hygiene was given to all patients, regardless of the group, in order not to bias data collection. However, higher levels of PI were observed after 3 months of periodontal treatment only for the SM group. One hypothesis is that smokers may be less motivated to keep a high-quality oral hygiene throughout the period of study^26^. Due to plaque accumulation, smokers showed a higher GI mean over 3 months. This finding is opposite to what is found in literature data^15^ ^,^ ^27^, in which inflammatory levels generally are reduced in smokers by the influence of the vasoconstrictor effect of nicotine, regardless of the plaque index^28^. In addition, a significant reduction in the periodontal parameters PPD and CAL was detected for both groups, SM and NS, after periodontal treatment. However, SM showed lower reduction in probing depth and gain in clinical attachment in comparison with NS. The same was observed in other studies^26^ ^,^ ^29^ ^,^ ^30^. Trombelli and Scabbia^29^ (1997) found that SM with furcation problems exhibited a less favorable healing pattern after surgery, caused by the interference of nicotine on collagen synthesis and bone formation. Jansson and Hagstrom^26^ (2002) reported that SM have high risk of recurrence of periodontitis in the periodontal maintenance phase, and need more surgical intervention. In contrast, Zuabi, et al.^30^ (1999) observed a higher reduction in probing depth in SM (0.81+0.11 mm) compared to NS (0.5+0.08 mm), after conventional therapy, showing that the effect of supragingival plaque control and clinical signs of periodontitis is yet controversial when smoking habits are considered.

Many variations in the distribution of *P. gingivalis fimA* genotypes can be found in literature, depending on the population examined. However, one finding is recurrent in periodontal studies, namely, greater frequency of the *fimA* genotype II in patients with chronic periodontitis^16^ ^,^ ^20^ ^,^ ^31^. This determined that *fimA* genotypes I and II were the most prevalent at baseline for both NS and SM groups, thus confirming the frequency of these types of fimbriae in patients with chronic periodontitis^6^. In agreement with our results, Beikler, et al.^7^ (2003) found that predominant *fimA* genotypes in 26 *P. gingivalis* isolates from Caucasian patients with periodontitis were types I (25.5%), II (38.2%) and IV (18.6%), by using PCR and restriction analysis. However, there was no difference in the association of the various *fimA* genotypes and disease severity. On the contrary, Teixeira, et al.^29^ (2000) found higher levels of genotype IV (69.6%) than II (28%), and the proportion of genotypes was associated with increasing probing depth only for genotype II.

Our study also found that genotype II was higher in SM compared to NS. The greater virulence of genotypes I and II can be attributed to their adhesiveness and invasiveness, which are key determinants for *P. gingivalis* virulence. *FimA* genotype I and II microorganisms are more adhesive to salivary proteins than other types, and their binding abilities are related to the sequence similarity of fimbrillin proteins^20^. This explains why genotypes I and II were more prevalent in this study, as well as in other studies^31^ ^,^ ^32^. High relative risk (RR) for the presence of genotype II, followed by genotypes I and III, was observed for SM compared with NS, at baseline, confirming the importance of these two genotypes for the development of periodontal disease.

Some studies have established that *fimA* genotype IV is considered an important virulence factor for the pathogenesis of periodontal disease^6^ ^,^ ^32^. However, in our study, there was no detection of type IV in the groups, disagreeing with some authors, who found not only genotypes I and II, but also genotype IV among the most frequently detected^30^ ^,^ ^31^. It is important to emphasize that genotyping was performed exclusively among participants with chronic periodontitis, and there was a variation in *P. gingivalis* genotypes regarding other types of periodontitis, a distinction that could change the course and evolution of the pathology.

In this study, we selected samples of *P. gingivalis* from our previous study^33^, in which the authors detected *P. gingivalis* in 50% of SM and 70.8% of NS, both groups with periodontal disease. The high frequency of *P. gingivalis* in patients with periodontitis is according with the results of Amano, et al.^6^ (2000), which detected *P. gingivalis* in 87.1% of patients with periodontitis. Type II *fimA* is associated with deeper pockets, whereas genotypes III and V seem to be involved in periodontitis to a lesser extent^12^. The results of this study corroborated with these findings, because a strong correlation was found between probing depth (>5 mm) and presence of type II *fimA.* Likewise, when associations between the fimbriae were tested in SM and NS at baseline and after 3 months, statistical analysis revealed significant differences between SM and NS, when II and V *fimA* genotypes were associated. This may be attributed to deeper pockets in SM compared with NS. Results obtained by Darby, et al.^34^ (2005) and Lee, et al.^35^ (2012) corroborate these results.

One limitation of this study is the absence of biochemical validation of the smoking status. Self-reported smoking status can underestimate true smoking prevalence and quantity due to a variety of factors such as misunderstanding, intentional deception, embarrassment, denial, shame, etc., inducing socially desirable responses. Measurement of cotinine, a primary metabolite of nicotine, can be detected in urine, saliva or serum and could provide a reliable biochemical marker of smoking status and other tobacco use or exposure over a period of 2 to 3 days^36^.

## Conclusion

Within the limitation of the relatively small sample size, this study concluded that the most prevalent *P. gingivalis fimA* genotypes detected in periodontal participants were genotypes I and II. However, the presence of *fimA* genotype II was higher in SM. Periodontal treatment was effective in controlling periodontal disease and in reducing *P. gingivalis fimA* type II and V. The authors suggest that more longitudinal studies are necessary to establish whether genotypes of *P. gingivalis fimA* can be maintained for long periods of time, and whether they influence the evolution of periodontal disease over time.
